# Impact of CDKN2A/B, MTAP, and TERT Genetic Alterations on Survival in IDH Wild Type Glioblastomas

**DOI:** 10.1007/s12672-022-00590-2

**Published:** 2022-11-15

**Authors:** Eric J. Hsu, Jamie Thomas, Elizabeth A. Maher, Michael Youssef, Robert D. Timmerman, Zabi Wardak, Tu D. Dan, Toral R. Patel, Dat T. Vo

**Affiliations:** 1 Department of Radiation Oncology, UT Southwestern Medical Center, Dallas, TX USA; 2 Department of Neurological Surgery, UT Southwestern Medical Center, Dallas, TX USA; 3 Department of Internal Medicine, Division of Hematology and Oncology, UT Southwestern Medical Center, Dallas, TX USA; 4 Department of Neurology, UT Southwestern Medical Center, Dallas, TX USA

**Keywords:** Next generation sequencing, WHO CNS5, Glioblastoma, CDKN2A, TERT, Radiation therapy

## Abstract

**Purpose:**

Poor outcomes in IDH wild-type (IDHwt) glioblastomas indicate the need to determine which genetic alterations can indicate poor survival and guidance of patient specific treatment options. We sought to identify the genetic alterations in these patients that predict for survival when adjusting particularly for treatments and other genetic alterations.

**Methods:**

A cohort of 167 patients with pathologically confirmed IDHwt glioblastomas treated at our institution was retrospectively reviewed. Next generation sequencing was performed for each patient to determine tumor genetic alterations. Multivariable cox proportional hazards analysis for overall survival (OS) was performed to control for patient variables.

**Results:**

CDKN2A, CDKN2B, and MTAP deletion predict for worse OS independently of other genetic alterations and patient characteristics (hazard ratio [HR] 2.192, *p* = 0.0017). Patients with CDKN2A copy loss (HR 2.963, *p* = 0.0037) or TERT mutated (HR 2.815, *p* = 0.0008) glioblastomas exhibited significant associations between radiation dose and OS, while CDKN2A and TERT wild type patients did not. CDKN2A deleted patients with NF1 mutations had worse OS (HR 1.990, *p* = 0.0540), while CDKN2A wild type patients had improved OS (HR 0.229, *p* = 0.0723). Patients with TERT mutated glioblastomas who were treated with radiation doses < 45 Gy (HR 3.019, *p* = 0.0010) but not those treated with ≥ 45 Gy exhibited worse OS compared to those without TERT mutations.

**Conclusion:**

In IDHwt glioblastomas, CDKN2A, CDKN2B, and MTAP predict for poor prognosis. TERT and CDKN2A mutations are associated with worse survival only when treated with lower radiation doses, thus potentially providing a genetic marker that can inform clinicians on proper dose-fractionation schemes.

**Supplementary Information:**

The online version contains supplementary material available at 10.1007/s12672-022-00590-2.

## Introduction

Diffuse gliomas are the most common adult type primary intracranial tumor, accounting for more than 80% of all malignant brain tumors [1]. Recently, the fifth edition of the WHO Classification of Tumors of the Central Nervous System (WHO CNS5) has reclassified diffuse glioma subtypes with greater emphasis on genetic and molecular profiling [2]. Such reclassifications factor in the different aggressiveness and biology conferred by these genetic and molecular alterations, which provide better prognostic insight for clinicians. The interactive effects between the various genetic alterations and patient characteristics, treatments, and especially other genetic alterations on patient outcome have yet to be well defined, especially when patients are stratified based on the recent WHO CNS5 reclassifications. This is particularly vital for IDH wild type (IDHwt) glioblastoma patients, who exhibit 5 year survival rates of as low as 5% [3].

The most notable reclassification scheme for glioblastomas involves the presence or absence of IDH mutations in astrocytomas. This stems from the fact that despite potentially similar histology, IDH mutations in tumors confer a fundamentally different biology and improved prognosis compared to IDHwt diffuse astrocytomas [4, 5]. Such classifications can guide and improve reliability of treatment regimens for specific diffuse glioma subtypes. This is evidenced by RTOG 9802, which demonstrates that radiation plus procarbazine, lomustine, and vincristine over radiation alone provides an overall survival benefit in IDHmut gliomas but not IDHwt glioblastomas [6]. Such an example defines the need to better characterize how the panel of other genetic alterations in IDHwt glioblastomas affect survival. It becomes vital to determine which genetic markers and gene interactions can define the need for intensification of therapy or addition of further adjuvant treatments in these patients. Therefore, in this study, we determine the genetic alteration profile in IDHwt glioblastoma patients. We then assess the impact of the most frequently altered genes on patient survival, adjusting for patient characteristics, treatments, and presence or absence of other gene alterations.

## Materials and methods

A database of 167 patients with IDHwt glioblastomas who were treated with neurological surgical intervention and/or radiation at our institution between April 2014 and December 2021 was retrospectively reviewed. Patients underwent pathological typing according to the 2021 WHO Classification of Tumors of the Central Nervous System [2]. The study was approved by the UT Southwestern institutional review board (IRB number STU 062014-027).

Patients received neurological surgical intervention in the form of total resection, subtotal resection, or stereotactic biopsy only. Tumor specimens were sent to Tempus laboratories for Next Generation Sequencing (NGS) to assess for genetic alterations of biological significance in the tumor. A targeted panel of 648 genes (Tempus xT) was selected for detection. MGMT promoter methylation was detected using MGMT specific PCR testing.

Patients received adjuvant radiation therapy targeted to the post-tumor resection cavity with doses ranging from 24 to 60.4 Gy in 4–33 fractions. Patients underwent CT simulation with a tailored head-thermoplastic mask in the supine position. A gross tumor volume (GTV) is delineated using a fused postoperative MRI on the T1 and T2 FLAIR sequences, followed by a creation of a clinical target volume (CTV) to cover the potential areas of microscopic disease. Then, a planning target volume (PTV) expansion was created to account for daily uncertainty in daily set-up and treatment delivery, per our institutional protocol and standards. All patients received concurrent temozolomide unless clinically contraindicated. Adjuvant temozolomide was typically initiated 4–6 weeks after surgery or radiation.

We evaluated patient pre-operative tumor size, defined as the largest dimension of the tumor on the most recent pre-operative MRI, and whether the tumor was multifocal, defined as having at least two separate lesions observed on MRI. We then assessed the patterns of failure, including in-field failures (within the 95% isodose volume), out-of-field failures, or marginal failures (within the 50–95% isodose volume) as observed radiographically on MRI. Time to recurrence was defined as the time from the end of the radiation treatment period to the first radiographic evidence of recurrence.

### Statistics

Overall survival (OS) and progression-free survival (PFS) were estimated using Kaplan–Meier method. Patients who were alive without evidence of recurrence were censored at the date of last follow up. *p* values were calculated from incidence of recurrence or death and survival curves were created with Cox proportional hazards tests. *p* values were considered significant at < 0.05.

Univariate and multivariable cox proportional hazards regression methods were used to determine the impact of patient covariates on OS as described previously by our group [7]. Hazard ratios and confidence intervals were calculated for each variable. Age, tumor size, and tumor mutational burden were analyzed as continuous variables, with the remaining variables being analyzed as categorical variables. Multivariable Cox proportional hazards regression models were used to adjust for patient characteristics, treatment regimens, and the most common genes detected by NGS for each multivariable analysis. The correlation matrix was calculated by normalizing covariate matrix calculated by the Pearson’s correlation of coefficients between each gene pair.

## Results

Our study included 167 patients with IDHwt glioblastomas at our institution who received NGS. Patient characteristics, including age, BMI, gender, radiation treatment details, surgical resection status, and tumor characteristics, are displayed (Table 1). All patients were treated with radiation therapy in 4–33 fractions, with increasing fractions corresponding to increased total radiation dose prescribed. Median follow-up for all patients was 11.5 months (range 0.2–99.2 months).

**Table 1:** Patient characteristics

Characteristics	Full cohort
Total patients	167
*Age (years)*	
Median	63.9
Range	24.5–85.1
*BMI*	
Median	27.2
Range	14.2–44.4
Gender	
*Male*	101
Female	66
*Karnofsky performance status*	
≥ 80	92
< 80	75
*Dose (Gy)*	
Median	60.0
Range	24.0–60.4
*Number of fractions*	
Median	30
Range	4–33
*Total surgical resection status*	
Positive	113
Negative	54
*Tumor size (mm)*	
Median	39
Range	7 – 82
*Multifocal disease*	
Positive	67
Negative	100
*Tumor mutational burden*	
Median	2.1
Range	0.5–23.3
*MGMT methylation status*	
Wild type	12
Methylated	42
*Follow up duration (months)*	
Median	11.5
Range	0.2–99.2

Gy, gray; BMI, body mass index

**Fig. 1:**
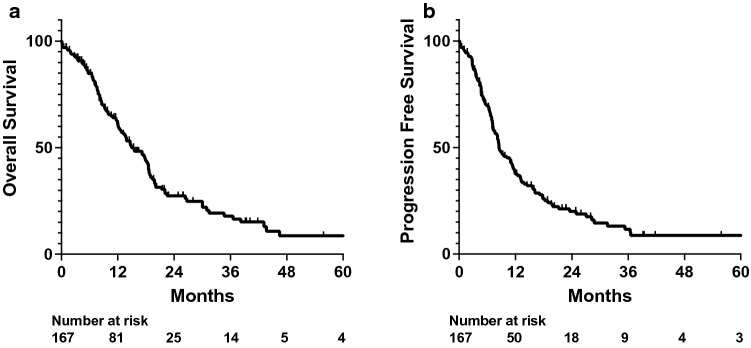
OS and PFS outcomes. Kaplan–Meyer plot of (**a**) overall survival (OS) and (**b**) progression free survival (PFS) for the full cohort of IDHwt glioblastoma patients

Median OS and PFS for the full cohort of patients were 15.4 and 8.5 months, respectively. 2-year OS and PFS were respectively 27.3 and 20.0% in this cohort (Fig. 1). NGS was performed on all patients, and patients were found to have alterations in 77 different genes, respectively (Additional file 1: Table S1). Gene alterations detected by NGS sequencing that were evaluated in our analyses included TERT, CDKN2A, CDKN2B, MTAP, TP53, NF1, CDK4, EGFR vIII, and PIK3CA, which were the genes with the most observed alterations in this cohort.

**Fig. 2:**
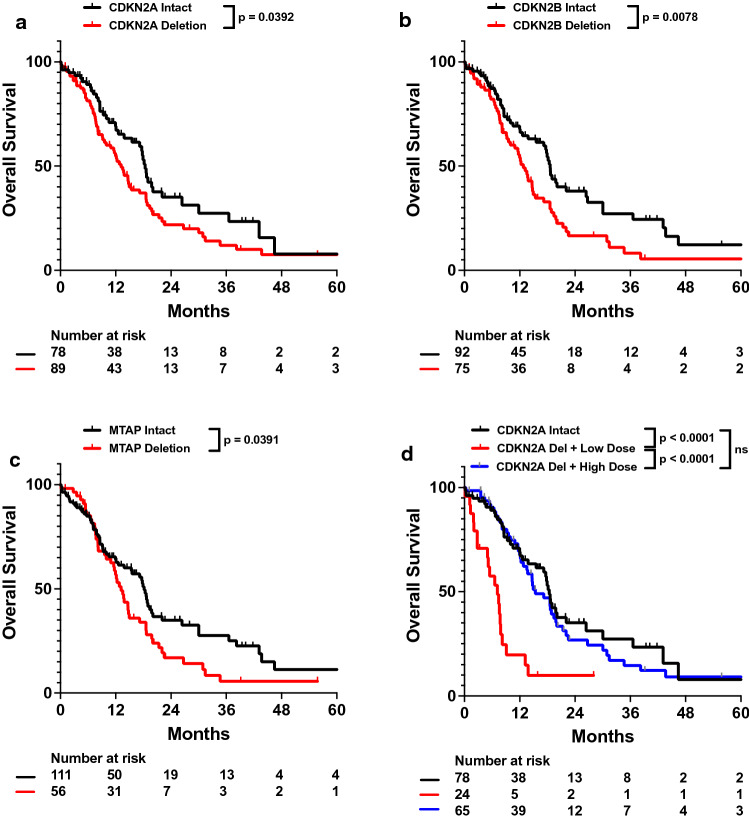
Univariate impact of CDKN2A homozygous deletion, CDKN2B homozygous deletion, MTAP homozygous deletion, on OS in full patient cohort. Kaplan–Meyer plots of OS comparing patients with different (**a**) CDKN2A, (**b**) CDKN2B, or (**c**) MTAP are shown. (**d**) Plot of OS comparing patients receiving at least 45 Gy radiation (labeled as High Dose) or less than 45 Gy radiation (labeled as Low Dose) with different CDKN2A deletion status is also shown. Statistical analysis was performed using Cox Proportional Hazards tests. OS, overall survival

Univariate analysis of patient characteristics was performed to determine genetic predictors of OS. From the NGS-detected genes assessed, CDKN2A, CDKN2B, and MTAP deletion were all associated with worse survival (Table 2; Fig. 2a–c). Notably, all patients who had CDKN2B or MTAP copy loss also had CDKN2A deletions, resulting in significant correlation between the three variables (Additional file 1: Fig. S1). After adjusting for patient characteristics, treatments, and tumor alterations, CDKN2A homozygous deletion was associated with worse OS (adj. hazard ratio [HR] 2.192, 95% CI 1.343–3.578; Table 3). Similar survival outcomes were observed when instead incorporating CDKN2B (adj. HR 2.062, 95% CI 1.279–3.326) or MTAP (adj. HR 2.101, 95% CI 1.275–3.463) deletion into a corresponding Cox Proportional Hazards model. Furthermore, consistent with previous literature, MGMT methylation was associated with improved survival [8].

**Table 2:** Univariate analysis of impact of patient characteristics, treatments, and tumor genetics on OS in full patient cohort

Variable	Hazard ratio	95% CI	*p* value
Age	1.011	0.995–1.027	0.1841
KPS ≥ 80	0.972	0.959–0.986	7.7 × 10^–5^
Dose < 45 Gy	2.232	1.472–3.383	0.0002
Total resection	0.661	0.439–0.995	0.0472
Tumor size	1.005	0.993–1.017	0.4553
Multifocal disease	1.378	0.942–2.015	0.0985
Tumor mutational burden	1.075	0.989–1.168	0.0891
MGMT methylation	0.539	0.325–0.895	0.0169
TERT mutation	1.324	0.825–2.123	0.2446
CDKN2A deletion	1.500	1.018–2.212	0.0405
CDKN2B deletion	1.666	1.139–2.436	0.0085
MTAP deletion	1.492	1.018–2.188	0.0405
PTEN alteration	1.135	0.778–1.655	0.5104
EGFR gain	0.862	0.589–1.262	0.4445
TP53 alteration	1.029	0.682–1.551	0.8924
NF1 alteration	1.169	0.712–1.920	0.5370
CDK4 deletion	0.936	0.524–1.675	0.8249
EGFR vIII Ex 2–7 deletion	0.850	0.491–1.471	0.5610
PIK3CA alteration	1.480	0.855–2.562	0.1613

Cox proportional hazards regression was performed on univariate models on the listed variables. Hazard ratios for age, dose, number fractions, tumor size, and tumor mutational burden were calculated as continuous variables, while the remaining variables were calculated as categorical variables. OS, overall survival; Gy, gray; KPS, Karnofsky performance status; CI, confidence interval

**Table 3:** Multivariable analysis of impact of patient characteristics, treatments, and tumor genetics on OS in full patient cohort

Variable	Hazard ratio (Adj.)	95% CI	*p* value
Age	1.005	0.986–1.024	0.6037
KPS ≥ 80	0.542	0.351–0.836	0.0056
Dose < 45 Gy	2.726	1.626–4.570	0.0001
Total resection	0.505	0.315–0.811	0.0047
Tumor size	1.003	0.989–1.016	0.6826
Multifocal disease	1.563	1.022–2.390	0.0392
Tumor mutational burden	1.065	0.971–1.168	0.1832
MGMT methylation	0.363	0.207–0.638	0.0004
TERT mutation	1.566	0.937–2.618	0.0869
CDKN2A deletion	2.192	1.343–3.578	0.0017
PTEN alteration	1.182	0.747–1.870	0.4762
EGFR gain	0.907	0.543–1.515	0.7079
TP53 alteration	1.110	0.679–1.817	0.6767
NF1 alteration	1.085	0.607–1.937	0.7838
CDK4 deletion	1.482	0.716–3.067	0.2887
EGFR vIII Ex 2–7 deletion	0.694	0.337–1.430	0.3219
PIK3CA alteration	1.238	0.625–2.450	0.5405

Cox proportional hazards regression was performed on multivariable models on the listed variables. Hazard ratios for age, dose, number fractions, tumor size, and tumor mutational burden were calculated as continuous variables, while the remaining variables were calculated as categorical variables. OS, overall survival; Gy, gray; KPS, Karnofsky performance status; CI, confidence interval

**Fig. 3:**
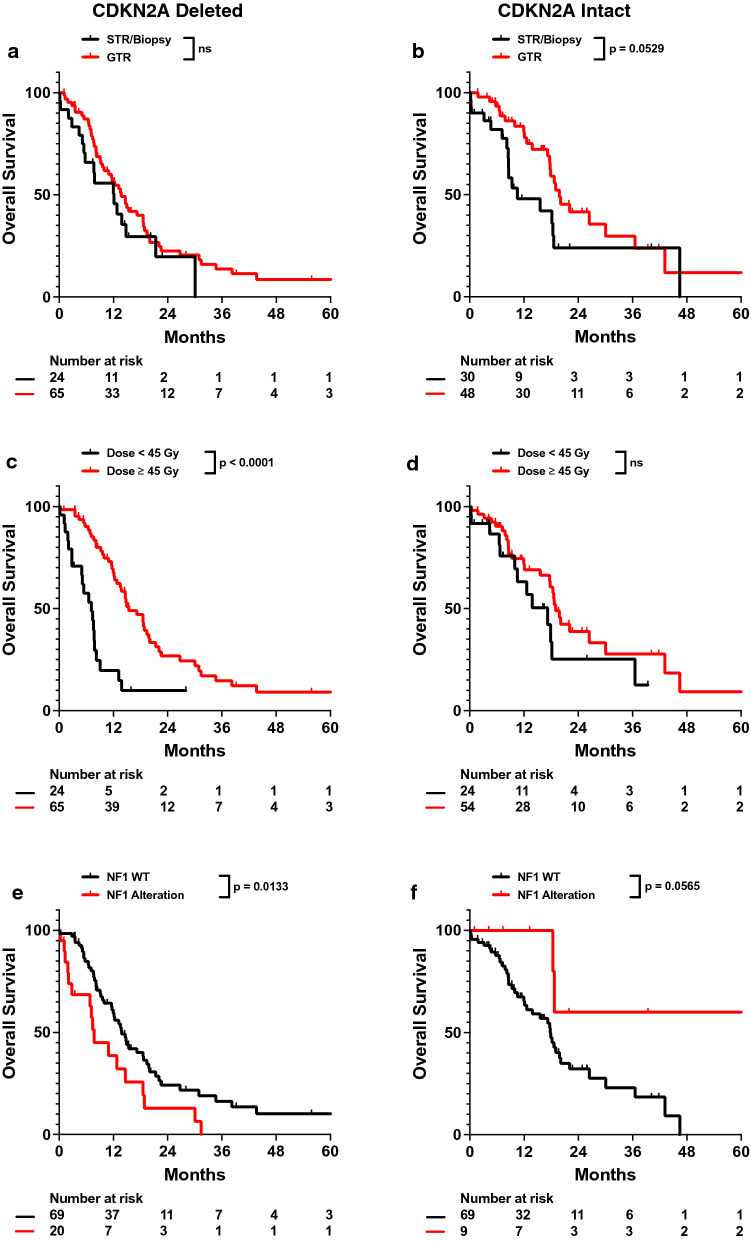
Univariate impact of total resection, radiation dose, and NF1 alteration on OS in CDKN2Adel and CDKN2Aint cohorts. Kaplan–Meyer plots of OS comparing patients with different (**a, b**) total resection status, (**c, d**) radiation dose, or (**e, f**) NF1 alteration status in CDKN2Adel and CDKN2Aint patients are shown. Statistical analysis was performed using Cox Proportional Hazards tests. OS, overall survival; GTR, gross total resection; STR, subtotal resection

As CDKN2A was the more frequently deleted in our cohort compared to CDKN2B and MTAP, we conducted further investigation only on CDKN2A. To better characterize the effects of CDKN2A homozygous deletion on survival, we performed subgroup analysis of IDHwt glioblastoma patients with CDKN2A copy deletions (CDKN2Adel) and with intact CDKN2A genes (CDKN2Aint). Patient characteristics between these two subgroups are relatively well balanced except for KPS (Additional file 1: Table S2). In CDKN2Adel patients, decreased radiation dose was associated with worse OS and total resection was not associated with improved OS (adj. HR 2.963, 95% CI 1.422–6.172). Notably, patients with CDKN2Adel tumors who received less than 45 Gy of radiation exhibited worse outcomes than those with either CDKN2Aint tumors or CDKN2Adel tumors but received more than 45 Gy radiation (Fig. 2d). In contrast, CDKN2Aint patients who received total resection of their tumors exhibited improved OS (adj. HR 0.392, 95% CI 0.166–0.925), but increased radiation dose was not associated with survival (Fig. 3a–d; Table 4; Additional file 1: Table S3). Univariate and multivariable analysis also demonstrated worse survival in CDKN2Adel patients with NF1 alterations (adj. HR 1.990, 95% CI 0.988–4.008). However, NF1 alterations were associated with improved OS in CDKN2Aint patients (adj. HR 0.229, 95% CI 0.046–1.143; Fig. 3e, f; Table 4; Additional file 1: Table S3).

**Table 4:** Multivariable analysis of impact of patient characteristics, treatments, and tumor genetics on OS in CDKN2Adel and CDKN2Aint IDHwt glioblastoma patients

Variable	CDKN2Adel			CDKN2Aint		
	Hazard ratio (Adj.)	95% CI	*p* value	Hazard ratio (Adj.)	95% CI	*p* value
Age	1.014	0.982–1.047	0.3918	1.028	0.998–1.058	0.0688
KPS ≥ 80	0.644	0.359–1.153	0.1385	0.359	0.143–0.900	0.0289
Dose < 45 Gy	2.963	1.422–6.172	0.0037	1.786	0.672–4.744	0.2447
Total resection	0.563	0.283–1.120	0.1014	0.392	0.166–0.925	0.0325
Tumor size	0.997	0.979–1.016	0.7642	1.007	0.986–1.028	0.5120
Multifocal disease	1.932	1.066–3.503	0.0300	1.135	0.543–2.373	0.7371
Tumor mutational burden	1.128	1.001–1.270	0.0474	0.989	0.805–1.214	0.9129
MGMT methylation	0.458	0.221–0.948	0.0353	0.202	0.062–0.662	0.0082
TERT mutation	1.464	0.655–3.269	0.3528	2.792	1.052–7.407	0.0391
PTEN alteration	1.480	0.809–2.706	0.2034	0.799	0.342–1.866	0.6045
EGFR gain	0.796	0.359–1.766	0.5751	0.874	0.363–2.102	0.7635
TP53 alteration	1.689	0.705–4.045	0.2395	0.833	0.377–1.839	0.6509
NF1 alteration	1.990	0.988–4.008	0.0540	0.229	0.046–1.143	0.0723
CDK4 deletion	6.243	1.156–33.72	0.0333	0.892	0.362–2.199	0.8034
EGFR vIII Ex 2–7 deletion	1.095	0.460–2.609	0.8369	0.149	0.014–1.648	0.1206
PIK3CA alteration	1.572	0.677–3.650	0.2921	0.496	0.089–2.750	0.4225

Cox proportional hazards regression was performed on multivariable models on the listed variables. Hazard ratios for age, dose, number fractions, tumor size, and tumor mutational burden were calculated as continuous variables, while the remaining variables were calculated as categorical variables. OS, overall survival; Gy, gray; KPS, Karnofsky performance status; CI, confidence interval

**Fig. 4:**
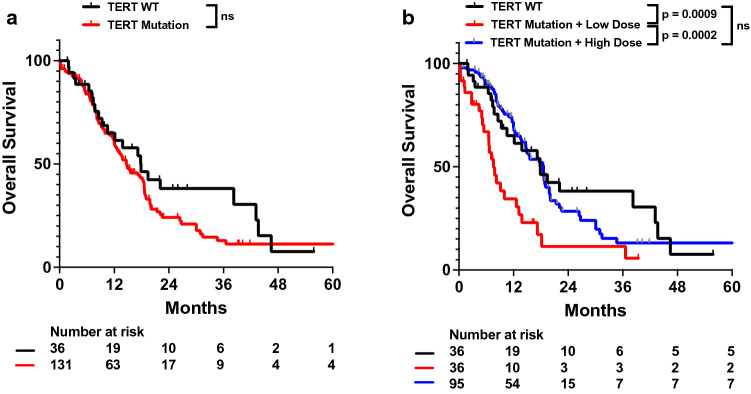
Univariate impact of TERT mutations on OS in full patient cohort. Kaplan–Meyer plots of OS comparing patients with (**a**) different TERT mutation status and (**b**) TERT wild type status vs TERT mutant status with patients receiving at least 45 Gy radiation (labeled as High Dose) vs TERT mutant status with patients receiving less than 45 Gy radiation (labeled as Low Dose). Statistical analysis was performed using Cox Proportional Hazards tests. OS, overall survival; WT, wild type

Mutations in TERT on univariate analysis were not associated with worse survival (HR 1.324, 95% CI 0.825–2.123; Table 2; Fig. 4a). However, on multivariable analysis, TERT mutations more prominently trended towards worse OS (adj. HR 1.566, 95% CI 0.937–2.618; Table 3). To elucidate this increase in significance from univariate to multivariable analysis, we further investigated how TERT may potentially interact with other variables incorporated into our multivariable model. We used Cox proportional hazard models to determine the impact of TERT mutations on OS when adjusting only for single patient characteristics. We observed that adjusting for radiation dose most affected the association between TERT mutations and patient survival (Additional file 1: Table S4). As TERT mutations have been observed to affect tumor cell sensitivity to DNA damage (i.e., from radiation or chemotherapy), we hypothesized whether there may be an interaction between TERT mutations and radiation dose response [9]. We thus assessed whether patients with TERT mutations who received lower doses of radiation exhibited worse survival. In both univariate and multivariable analysis, patients who had TERT mutations and received more than 45 Gy of radiation did not exhibit significant differences in survival compared to those without TERT mutations. However, those with TERT mutations but received less than 45 Gy of radiation exhibited significantly worse OS (adj. HR 3.019, 95% CI 1.563–5.831; Fig. 4b; Table 4).

**Fig. 5:**
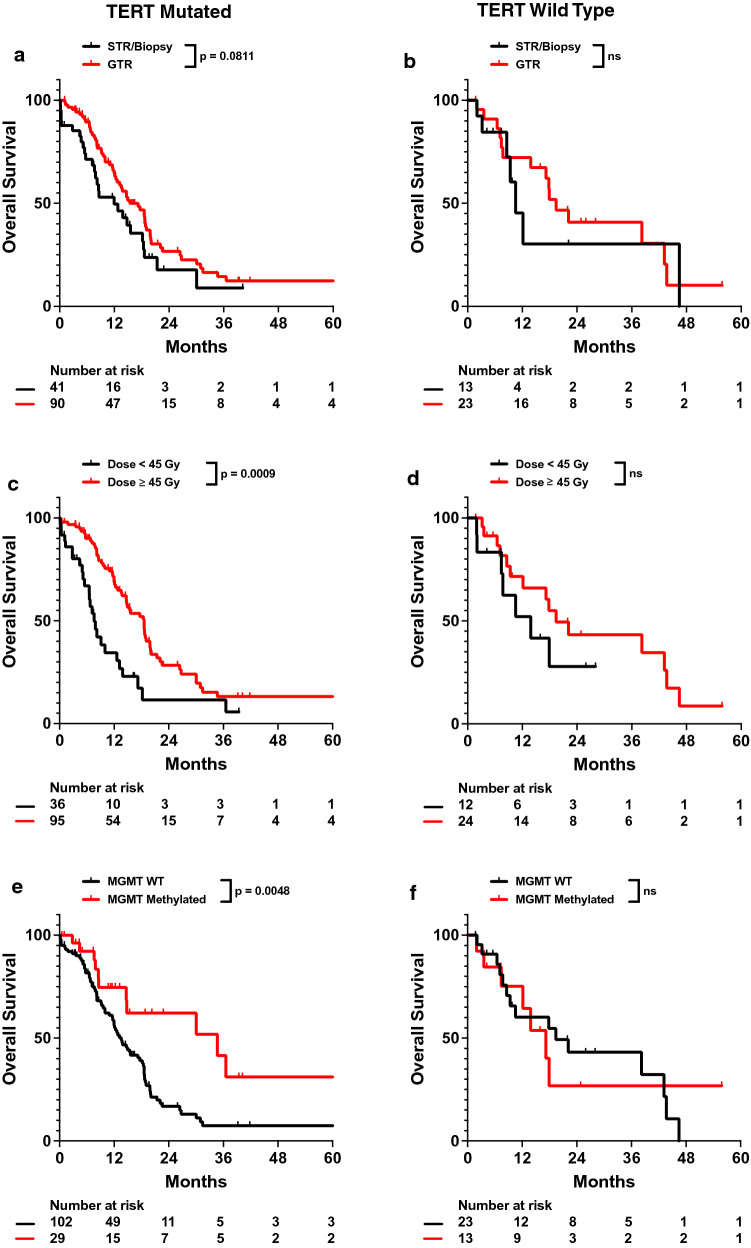
Univariate impact of total resection, radiation dose, and MGMT methylation on OS in TERTmut and TERTwt cohorts Kaplan–Meyer plots of OS comparing patients with different (**a, b**) total resection status, (**c, d**) radiation dose, or (**e, f**) MGMT methylation status in TERTmut and TERTwt patients are shown. Statistical analysis was performed using Cox Proportional Hazards tests. OS, overall survival; GTR, gross total resection; STR, subtotal resection; WT, wild type

To better evaluate the impact of TERT mutations on survival, we assessed which patient characteristics were associated with survival in TERTmut and TERTwt IDHwt glioblastoma patient cohorts. Patient characteristics of each subgroup are relatively well balanced (Additional file 1: Table S5). In TERTmut patients, total resection (adj. HR 0.401, 95% CI 0.228–0.704) was associated with improved OS and decreased radiation dose (adj. HR 2.815, 95% CI 1.533–5.171) was associated with worse OS. However, these associations were not observed in TERTwt patients (Fig. 5a–d; Table 6; Additional file 1: Table S6). MGMT methylation was also observed to be associated with improved OS (adj. HR 0.186, 95% CI 0.086–0.399) on both univariate and multivariable analysis in TERTmut patients but not TERTwt patients (Fig. 5; Table 6; Additional file 1: Table S6). Overall, other than significantly increased response to dose, prognostic indicators of TERTmut patient cohort generally matched up with those in the full patient cohort. However, many of these prognostic indicators were not associated with OS in TERTwt patients.

## Discussion

With the significant enhancement in the role of molecular diagnostics in the classification of CNS tumors, assessment of tumors and genetics by NGS becomes vital. In particular, as IDHwt glioblastomas are such prevalent adult brain tumors with continued poor prognosis, evaluating how commonly altered genetic profiles in these tumors affect survival can guide further indications for adjuvant therapies. In this study, we investigated which gene alterations were detected by NGS in patients with IDHwt glioblastomas and how they affected survival. Furthermore, we assessed how these genetic impacts on survival can be affected by radiation dose and treatment regimens.

Our full population included 167 glioblastoma patients, all of whom received NGS. Our patients exhibited overall survival outcomes consistent with other studies (Fig. 1) [3]. The most common genes that were found to be altered in our patient cohort were consistent with the typical characteristic genetic profiles found in IDHwt glioblastoma patients as per the 2021 WHO Classification guidelines (Additional file 1: Table S1) [2].

For IDHwt glioblastomas, 2021 WHO Classification identifies that TERT promoter mutations and EGFR alterations are characteristically found. Our gene list in these patients also identified TERT and EGFR as commonly altered genes, along with CDKN2A/B, PTEN, MTAP, and TP53, all of which were altered in over 30% of patients. Of these genes, while EGFR amplification and CDKN2A/B deletions have been previously observed to be associated with worse survival, the prognostic significance of TERT, PTEN, MTAP, and TP53 are unclear [10–16]. In our cohort, after adjusting for patient characteristics, treatments, and other tumor genetic alterations, we observed that while CDKN2A and CDKN2B deletions were associated with worse survival, EGFR amplification was not correlated with poor outcomes (Fig. 2; Table 3). We also identified MTAP deletion, which was often co-deleted with CDKN2A, as a predictor of worse OS (Fig. 2; Table 3; Additional file 1: Fig. S1). The prognostic significance of MTAP deletion has not been previously well characterized, but our results suggest that MTAP deletion may potentially serve as a surrogate marker for CDKN2A deletion in prognostic evaluation of IDHwt glioblastoma patients [17].

We sought to further assess how CDKN2A homozygous deletion affected patient outcomes and thus analyzed CDKN2Adel and CDKN2Aint patient subgroups. We observed that CDKN2Adel patients exhibited improved outcomes when treated with higher doses of radiation, but not necessarily total tumor resection. In contrast, CDKN2Aint patients exhibited the opposite trend—improved survival was associated with total tumor resection, increased radiation dose (Fig. 3a–d; Table 4). Biologically, CDKN2A in tumor cells halts progression of the cell proliferation cycle at the G1 phase [18, 19]. As cells proliferating through the G2 phase of the cell cycle exhibit increased sensitivity to radiation, suppression of CDKN2A may increase the population of cells proceeding from the G1 phase into the G2 phase, which would improve radiosensitivity in CDKN2Adel tumors [20]. As CDKN2A increases tumor cell proliferation, loss of this tumor suppressor gene increases proliferation and invasiveness [21]. Such invasiveness observed in CDKN2Adel patients may not be easily evident during neurosurgical intervention, which may contribute to the lack of association between total resection and survival in CDKN2Adel patients. CDKN2A loss has also been observed to drive NF1 associated malignant transformation in neurofibromas, which may suggest an interaction between CDKN2A and NF1 in other tumor histologies as well that can potentially affect patient outcome (Fig. 3e, f; Table 4) [22]. It is unclear how NF1 could provide a protective prognostic effect in CDKN2Aint patients, but this dichotomous impact of NF1 on survival in CDKN2A stratified patients suggests that CDKN2A may significantly modulate NF1 activity in IDHwt glioblastoma patients. Further clinical studies patients with NF1 alterations in CDKN2Aint patients and mechanistic studies would need to be assessed further elucidate this observation.

While TERT mutations are one of the characteristic mutations in IDHwt glioblastomas, their prognostic significance remains controversial. Some previous studies identify TERT mutation status as an independent predictor of poor survival in IDHwt glioblastoma patients, while others find that it is confounded by other genes [12, 13]. We found that while TERT mutations were not significant prognostic indicators on univariate analysis, they were more associated with worse survival on multivariable analysis (Tables 2, 3). Such a result could be due to biases contributed by omitted or suppressor variables during univariate analysis, so we sought to determine which variables may be affecting the impact of TERT mutations on survival. TERT mutations result in increased TERT reactivation and telomerase activity, which results in a more active DNA damage response and reduced sensitivity to DNA damaging treatments such as radiation [9, 23, 24]. Thus, patients with TERT mutations may require higher doses of radiation to achieve improved survival outcomes. Indeed, we observe that in TERTmut patients compared to TERTwt patients, only those who received lower doses of radiation had worse OS (Fig. 4; Table 5).

**Table 5:** Multivariable analysis of impact of TERT mutation status and radiation dose on OS in full patient cohort

Variable	Hazard ratio (Adj.)	95% CI	*p* value
TERT mutation + high dose	1.150	0.672–1.970	0.6097
TERT mutation + low dose	3.019	1.563–5.831	0.0010
Age	1.010	0.992–1.028	0.2832
KPS ≥ 80	0.566	0.366–0.875	0.0105
Total resection	0.518	0.323–0.832	0.0065
Tumor size	1.003	0.990–1.017	0.6440
Multifocal disease	1.476	0.969–2.250	0.0699
Tumor mutational burden	1.079	0.983–1.185	0.1103
MGMT methylation	0.372	0.211–0.655	0.0006
CDKN2A deletion	2.239	1.362–3.681	0.0015
PTEN alteration	1.152	0.727–1.825	0.5469
EGFR gain	0.905	0.544–1.506	0.7011
TP53 alteration	1.235	0.759–2.011	0.3961
NF1 alteration	1.096	0.611–1.966	0.7576
CDK4 deletion	1.327	0.636–2.768	0.4510
EGFR vIII Ex 2–7 deletion	0.714	0.348–1.468	0.3601
PIK3CA alteration	1.300	0.655–2.580	0.4533

Cox proportional hazards regression was performed on multivariable models on the listed variables. Hazard ratios for age, dose, number fractions, tumor size, and tumor mutational burden were calculated as continuous variables, while the remaining variables were calculated as categorical variables. High Dose is considered as at least 45 Gy radiation while Low Dose is considered as less than 45 Gy radiation. OS, overall survival; Gy, gray; KPS, Karnofsky performance status; CI, confidence interval

To better evaluate the effects of TERT on radiation and survival, we analyzed TERTmut and TERTwt IDHwt glioblastoma subgroups. We observed that TERTmut IDHwt glioblastoma patients exhibited significantly improved survival in response to total resection, higher doses of radiation, and MGMT methylation. In contrast, none of these variables affected patient outcomes in TERTwt IDHwt glioblastoma patients (Fig. 5; Table 6). Previous studies have observed that MGMT methylation may potentially modulate TERT mutation effects on survival, which is consistent with the lack of sensitivity of only TERTwt patients to MGMT methylation in our study [25]. The lack of radiation dose response in TERTwt patients but not TERTmut patients also suggests that observations of dose response sensitivity in IDHwt glioblastomas may be dependent on particular genetic alterations, such as CDKN2A and TERT. Our results thus suggest that CDKN2A and TERT mutations may be predictors for poor survival that may be salvaged by higher doses of radiation. This may provide clinicians with a genetic marker indicating potential need for dose-fractionation schemes with higher total or biologically effective doses or at the minimum a lack of dose de-escalation.

**Table 6:** Multivariable analysis of impact of patient characteristics, treatments, and tumor genetics on OS in TERTmut and TERTwt IDHwt glioblastoma patients

Variable	TERTmut			TERTwt		
	Hazard ratio (Adj.)	95% CI	*p* value	Hazard ratio (Adj.)	95% CI	*p* value
Age	1.023	0.996–1.051	0.0942	1.009	0.961–1.059	0.7136
KPS ≥ 80	0.721	0.416–1.249	0.2437	0.326	0.071–1.487	0.1476
Dose < 45 Gy	2.815	1.533–5.171	0.0008	2.574	0.550–12.060	0.2301
Total resection	0.401	0.228–0.704	0.0015	0.217	0.032–1.451	0.1150
Tumor size	1.015	0.999–1.033	0.0715	1.017	0.947–1.092	0.6486
Multifocal disease	1.811	1.094–2.998	0.0209	0.469	0.052–4.229	0.4997
Tumor mutational burden	1.057	0.929–1.203	0.4008	0.986	0.707–1.375	0.9327
MGMT methylation	0.186	0.086–0.399	1.6 × 10^–5^	0.957	0.237–3.861	0.9504
CDKN2A deletion	3.225	1.689–6.157	0.0004	3.563	0.641–19.798	0.1465
PTEN alteration	1.118	0.655–1.906	0.6827	0.930	0.184–4.697	0.9299
EGFR gain	1.052	0.569–1.946	0.8716	3.747	0.321–43.758	0.2921
TP53 alteration	1.941	0.996–3.783	0.0513	0.424	0.045–3.995	0.4536
NF1 alteration	0.912	0.464–1.791	0.7886	11.601	0.709–189.83	0.0857
CDK4 deletion	1.812	0.792–4.147	0.1593	4.691	0.507–43.396	0.1733
EGFR vIII Ex 2–7 deletion	0.588	0.264–1.309	0.1934	1.709	0.169–17.259	0.6496
PIK3CA alteration	1.428	0.652–3.128	0.3733	6.787	0.224–205.45	0.2710

Cox proportional hazards regression was performed on multivariable models on the listed variables. Hazard ratios for age, dose, number fractions, tumor size, and tumor mutational burden were calculated as continuous variables, while the remaining variables were calculated as categorical variables. OS, overall survival; Gy, gray; KPS, Karnofsky performance status; CI, confidence interval

Our study had the traditional limitations that are relevant to all retrospective evaluations. These weaknesses include non-random treatment group allocation, selection bias, and non-random loss to follow up intrinsic to any non-randomized non-prospective study [26]. Despite this, our study still accounts for loss to follow up during statistical analysis. Secondly, not all patients with IDHwt glioblastomas received NGS, which may result in some selection bias. Lastly, because NGS is a more recently implemented technology, using it as a step in diagnostic workup of IDHwt glioblastoma patients results in a patient population with more recent diagnosis and treatment. This results in decreased observations of shorter-term mortality.

Taken together, our study provides a real-world analysis of NGS detected genetic profiles in IDHwt glioblastoma patients. In our patient cohort, we identified CDKN2A, CDKN2B, and MTAP as predictors of poor survival that are independent of other genetic alterations and patient characteristics. Patients with CDKN2A copy deletion exhibited improved survival with higher doses of radiation, but not those without CDKN2A loss. CDKN2A mutations may also potentially modulate NF1 activity, which ultimately affects patient outcome. We further observe that TERT mutations correspond with worse patient outcomes when patients receive lower doses of radiation but not when patients are treated with higher radiation doses. These results can inform clinicians on which genetic markers indicate a need for further adjuvant treatment, in particular intensification of radiation in the setting of CDKN2A or TERT mutations.

## Supplementary Information

**Additional file 1. Additional supplementary figures and tables as referenced in the manuscript text**

## Data Availability

The datasets generated during and/or analyzed during the current study are available from the corresponding author on reasonable request.
