# Supplementary material for: Impact of CDKN2A/B, MTAP, and TERT Genetic Alterations on Survival in IDH Wild Type Glioblastomas
Source: Discov Oncol. 2022 Nov 15;13:126. doi: 10.1007/s12672-022-00590-2 (PMC9666584; doi:10.1007/s12672-022-00590-2)
Supplement: Supplementary file 1 — Additional file 1. Additional supplementary figures and tables as referenced in the manuscript text [file 12672_2022_590_MOESM1_ESM.docx]

**Supplementary Data**

**Supplementary Table 1.** All gene alterations detected by next generation sequencing and frequency in 167 IDHwt glioblastoma patients.

**Supplementary Figure 1.** Correlation matrix for most frequent Next Generation Sequence discovered genes in IDHwt glioblastoma patients. Pearson’s R Coefficient of Correlation was calculated for alterations between each pair of genes. Each pair’s correlation coefficient is shown in their corresponding box.

**Supplementary Table 2.** Patient Characteristics in CDKN2Adel and CDKN2Aint cohorts. Abbreviations: Gy = Gray, BMI = Body Mass Index.

**Supplementary Table 3.** Univariate analysis of impact of patient characteristics, treatments, and tumor genetics on OS in CDKN2Adel and CDKN2Aint IDHwt glioblastoma patients. Cox Proportional Hazards Regression was performed on univariate models on the listed variables. Hazard ratios for age, dose, number fractions, tumor size, and tumor mutational burden were calculated as continuous variables, while the remaining variables were calculated as categorical variables. Abbreviations: OS = Overall Survival, Gy = Gray, KPS = Karnofsky Performance Status, CI = Confidence Interval.

**Supplementary Table 4.** Bivariate analysis of TERT mutation impact on OS in IDHwt glioblastoma patients with adjustment for listed patient characteristics, treatments, and tumor genetics. Cox Proportional Hazards Regression was performed on bivariate models including TERT mutations and the listed variables. Hazard ratios, confidence intervals, and p values for TERT mutations in each model is shown. Abbreviations: OS = Overall Survival, Gy = Gray, KPS = Karnofsky Performance Status, CI = Confidence Interval.

**Supplementary Table 5.** Patient Characteristics in TERTmut and TERTwt cohorts. Abbreviations: Gy = Gray, BMI = Body Mass Index.

**Supplementary Table 6.** Univariate analysis of impact of patient characteristics, treatments, and tumor genetics on OS in TERTmut and TERTwt IDHwt glioblastoma patients. Cox Proportional Hazards Regression was performed on univariate models on the listed variables. Hazard ratios for age, dose, number fractions, tumor size, and tumor mutational burden were calculated as continuous variables, while the remaining variables were calculated as categorical variables. Abbreviations: OS = Overall Survival, Gy = Gray, KPS = Karnofsky Performance Status, CI = Confidence Interval.

**Supplementary Table 1.** All gene alterations detected by next generation sequencing in 167 IDHwt glioblastoma patients.

| **Gene** | **Number/Frequency of Detected Alterations** | |  | **Gene** | **Number/Frequency of Detected Alterations** | |
| --- | --- | --- | --- | --- | --- | --- |
| TERT | 131 | 78.44% |  | YEATS4 | 2 | 1.20% |
| CDKN2A | 94 | 56.29% |  | ATR | 2 | 1.20% |
| CDKN2B | 75 | 44.91% |  | FBXO11 | 2 | 1.20% |
| PTEN | 74 | 44.31% |  | TOP1 | 2 | 1.20% |
| EGFR | 70 | 41.92% |  | SUZ12 | 2 | 1.20% |
| MTAP | 56 | 33.53% |  | FANCA | 2 | 1.20% |
| TP53 | 53 | 31.74% |  | HNF1A | 2 | 1.20% |
| NF1 | 29 | 17.37% |  | DAXX | 2 | 1.20% |
| CDK4 | 23 | 13.77% |  | DNMT3A | 2 | 1.20% |
| EGFR vIII | 21 | 12.57% |  | MLH1 | 1 | 0.60% |
| PIK3CA | 21 | 12.57% |  | NRAS | 1 | 0.60% |
| RB1 | 20 | 11.98% |  | PBRM1 | 1 | 0.60% |
| PDGFRA | 15 | 8.98% |  | WT1 | 1 | 0.60% |
| PIK3R1 | 14 | 8.38% |  | GATA3 | 1 | 0.60% |
| MDM4 | 11 | 6.59% |  | FOXO1 | 1 | 0.60% |
| PTPN11 | 11 | 6.59% |  | Focal | 1 | 0.60% |
| MDM2 | 9 | 5.39% |  | SBDS | 1 | 0.60% |
| EGFR vIVa | 6 | 3.59% |  | DDX5 | 1 | 0.60% |
| SETD2 | 5 | 2.99% |  | LZTR1 | 1 | 0.60% |
| STAG2 | 5 | 2.99% |  | JAK1 | 1 | 0.60% |
| KIT | 5 | 2.99% |  | KMT2A | 1 | 0.60% |
| ATRX | 4 | 2.40% |  | BCOR | 1 | 0.60% |
| BRAF | 4 | 2.40% |  | MAX | 1 | 0.60% |
| MSH6 | 4 | 2.40% |  | FOS | 1 | 0.60% |
| KDR | 4 | 2.40% |  | ZFHX3 | 1 | 0.60% |
| ERRFI1 | 3 | 1.80% |  | GLI1 | 1 | 0.60% |
| FRS2 | 3 | 1.80% |  | MLH3 | 1 | 0.60% |
| FGFR3 | 3 | 1.80% |  | MSH2 | 1 | 0.60% |
| IKZF1 | 3 | 1.80% |  | BRCA2 | 1 | 0.60% |
| H3F3A | 3 | 1.80% |  | MYCN | 1 | 0.60% |
| CDK6 | 3 | 1.80% |  | FAS | 1 | 0.60% |
| TSC1 | 2 | 1.20% |  | BCL6 | 1 | 0.60% |
| FGFR1 | 2 | 1.20% |  | STAT6 | 1 | 0.60% |
| AKT3 | 2 | 1.20% |  | EZH2 | 1 | 0.60% |
| CREBBP | 2 | 1.20% |  | CCND2 | 1 | 0.60% |
| CHEK2 | 2 | 1.20% |  | SOX2 | 1 | 0.60% |
| KDM6A | 2 | 1.20% |  | ARID2 | 1 | 0.60% |
| ATP7B | 2 | 1.20% |  | TET2 | 1 | 0.60% |
|  |  |  |  |  |  |  |

**Supplementary Figure 1.** Correlation matrix for most frequent Next Generation Sequence discovered genes in full patient cohort.

**Supplementary Table 2.** Patient Characteristics in CDKN2Adel and CDKN2Aint cohorts

| **Characteristics** | **CDKN2Adel** | **CDKN2Aint** |
| --- | --- | --- |
| **Total Patients** | 89 | 78 |
| **Age (years)** |  |  |
| **Median** | 63.9 | 64.1 |
| **Range** | 32.2 – 85.1 | 24.5 – 82.5 |
| **BMI** |  |  |
| **Median** | 27.0 | 27.5 |
| **Range** | 18.8 – 43.3 | 14.2 – 44.4 |
| **Gender** |  |  |
| **Male** | 48 | 48 |
| **Female** | 41 | 30 |
| **Karnofsky Performance Score** |  |  |
| **≥ 80** | 46 | 60 |
| **< 80** | 43 | 18 |
| **Dose (Gy)** |  |  |
| **Median** | 60.0 | 60.0 |
| **Range** | 25.0 – 60.0 | 24.0 – 60.4 |
| **Number of Fractions** |  |  |
| **Median** | 30 | 30 |
| **Range** | 5 – 33 | 4 – 33 |
| **Total Resection** |  |  |
| **Positive** | 65 | 48 |
| **Negative** | 24 | 30 |
| **Tumor Size (mm)** |  |  |
| **Median** | 39 | 41 |
| **Range** | 13 – 80 | 7 – 82 |
| **Multifocal Disease** |  |  |
| **Positive** | 36 | 31 |
| **Negative** | 53 | 47 |
| **Tumor Mutational Burden** |  |  |
| **Median** | 2.1 | 2.2 |
| **Range** | 0.5 – 19.6 | 0.8 – 23.3 |
| **MGMT Methylation Status** |  |  |
| **Wild Type** | 66 | 59 |
| **Methylated** | 23 | 19 |
| **Follow Up Duration (months)** |  |  |
| **Median** | 11.6 | 11.3 |
| **Range** | 0.2 – 99.2 | 0.2 – 97.1 |

**Supplementary Table 3.** Univariate analysis of impact of patient characteristics, treatments, and tumor genetics on OS in CDKN2Adel and CDKN2Aint IDHwt glioblastoma patients

|  | **CDKN2Adel** | | | **CDKN2Aint** | | |
| --- | --- | --- | --- | --- | --- | --- |
| **Variable** | **Hazard Ratio (Adj.)** | **95% CI** | **p value** | **Hazard Ratio (Adj.)** | **95% CI** | **p value** |
| **Age** | 1.019 | 0.994 - 1.044 | 0.1318 | 1.006 | 0.984 - 1.028 | 0.5855 |
| **KPS ≥ 80** | 0.975 | 0.959 - 0.992 | 0.0040 | 0.968 | 0.944 - 0.993 | 0.0110 |
| **Dose ≥ 45 Gy** | 3.500 | 2.001 - 6.120 | 1.1E-05 | 1.631 | 0.833 - 3.194 | 0.1539 |
| **Total Resection** | 0.721 | 0.411 - 1.263 | 0.2528 | 0.538 | 0.287 - 1.010 | 0.0536 |
| **Tumor Size** | 0.999 | 0.984 - 1.014 | 0.8512 | 1.012 | 0.992 - 1.033 | 0.2408 |
| **Multifocal Disease** | 1.453 | 0.896 - 2.357 | 0.1299 | 1.332 | 0.714 - 2.485 | 0.3677 |
| **Tumor Mutational Burden** | 1.109 | 1.023 - 1.201 | 0.0115 | 0.969 | 0.798 - 1.175 | 0.7468 |
| **MGMT Methylation** | 0.569 | 0.310 - 1.045 | 0.0691 | 0.455 | 0.177 - 1.169 | 0.1019 |
| **TERT Mutation** | 1.228 | 0.651 - 2.315 | 0.5261 | 1.366 | 0.663 - 2.815 | 0.3975 |
| **PTEN Alteration** | 1.272 | 0.787 - 2.056 | 0.3259 | 0.998 | 0.540 - 1.845 | 0.9957 |
| **EGFR Gain** | 0.640 | 0.394 - 1.040 | 0.0717 | 1.202 | 0.637 - 2.268 | 0.5705 |
| **TP53 Alteration** | 1.587 | 0.859 - 2.932 | 0.1406 | 1.033 | 0.559 - 1.911 | 0.9165 |
| **NF1 Alteration** | 2.021 | 1.158 - 3.527 | 0.0133 | 0.248 | 0.059 - 1.039 | 0.0565 |
| **CDK4 Deletion** | 2.810 | 0.671 - 11.76 | 0.1573 | 1.090 | 0.543 - 2.187 | 0.8090 |
| **EGFR vIII Exon 2-7 Deletion** | 0.704 | 0.389 - 1.276 | 0.2476 | 0.672 | 0.091 - 4.940 | 0.6959 |
| **PIK3CA Alteration** | 1.567 | 0.851 - 2.884 | 0.1493 | 0.804 | 0.193 - 3.356 | 0.7651 |

**Supplementary Table 4.** Bivariate analysis of TERT mutation impact on OS in IDHwt glioblastoma patients with adjustment for listed patient characteristics, treatments, and tumor genetics.

| **Variable** | **Hazard Ratio (Adj.)** | **95% CI** | **p value** |
| --- | --- | --- | --- |
| **Univariate Hazard Ratio** | 1.324 | 0.825 - 2.123 | 0.2446 |
| **Age** | 1.365 | 0.848 - 2.195 | 0.2001 |
| **KPS ≥ 80** | 1.373 | 0.855 - 2.206 | 0.1900 |
| **Dose ≥ 45 Gy** | 1.461 | 0.909 - 2.349 | 0.1173 |
| **Total Resection** | 1.333 | 0.830 - 2.139 | 0.2341 |
| **Tumor Size** | 1.328 | 0.828 - 2.132 | 0.2395 |
| **Multifocal Disease** | 1.294 | 0.806 - 2.079 | 0.2854 |
| **Tumor Mutational Burden** | 1.334 | 0.832 - 2.140 | 0.2313 |
| **MGMT Methylation** | 1.307 | 0.813 - 2.099 | 0.2686 |
| **CDKN2A Deletion** | 1.268 | 0.789 - 2.038 | 0.3267 |
| **PTEN Alteration** | 1.358 | 0.843 - 2.187 | 0.2080 |
| **EGFR Gain** | 1.323 | 0.825 - 2.122 | 0.2457 |
| **TP53 Alteration** | 1.323 | 0.823 - 2.125 | 0.2473 |
| **NF1 Alteration** | 1.307 | 0.812 - 2.102 | 0.2703 |
| **CDK4 Deletion** | 1.321 | 0.821 - 2.123 | 0.2512 |
| **EGFR vIII Exon 2-7 Deletion** | 1.345 | 0.837 - 2.161 | 0.2213 |
| **PIK3CA Alteration** | 1.308 | 0.816 - 2.099 | 0.2651 |

**Supplementary Table 5.** Patient Characteristics in TERTmut and TERTwt cohorts

| **Characteristics** | **TERTmut** | **TERTwt** |
| --- | --- | --- |
| **Total Patients** | 131 | 37 |
| **Age (years)** |  |  |
| **Median** | 63.4 | 67.6 |
| **Range** | 25.2 – 85.1 | 24.5 – 82.2 |
| **BMI** |  |  |
| **Median** | 27.4 | 26.3 |
| **Range** | 18.8 – 44.4 | 14.2 – 43.3 |
| **Gender** |  |  |
| **Male** | 82 | 19 |
| **Female** | 49 | 17 |
| **Karnofsky Performance Score** |  |  |
| **≥ 80** | 74 | 18 |
| **< 80** | 57 | 18 |
| **Dose (Gy)** |  |  |
| **Median** | 60.0 | 54.0 |
| **Range** | 24.0 – 60.0 | 25.0 – 60.4 |
| **Number of Fractions** |  |  |
| **Median** | 30 | 30 |
| **Range** | 4 – 33 | 5 – 33 |
| **Total Resection** |  |  |
| **Positive** | 90 | 23 |
| **Negative** | 41 | 13 |
| **Tumor Size (mm)** |  |  |
| **Median** | 38 | 41 |
| **Range** | 12 – 82 | 7 – 78 |
| **Multifocal Disease** |  |  |
| **Positive** | 57 | 10 |
| **Negative** | 74 | 26 |
| **Tumor Mutational Burden** |  |  |
| **Median** | 2.1 | 2.4 |
| **Range** | 0.5 – 16.7 | 0.8 – 23.3 |
| **MGMT Methylation Status** |  |  |
| **Wild Type** | 102 | 23 |
| **Methylated** | 29 | 13 |
| **Follow Up Duration (months)** |  |  |
| **Median** | 11.5 | 11.3 |
| **Range** | 0.2 – 99.2 | 0.6 – 55.8 |

**Supplementary Table 6.** Univariate analysis of impact of patient characteristics, treatments, and tumor genetics on OS in TERTmut and TERTwt IDHwt glioblastoma patients

|  | **TERTmut** | | | **TERTwt** | | |
| --- | --- | --- | --- | --- | --- | --- |
| **Variable** | **Hazard Ratio (Adj.)** | **95% CI** | **p value** | **Hazard Ratio (Adj.)** | **95% CI** | **p value** |
| **Age** | 1.018 | 0.997 - 1.039 | 0.0965 | 1.002 | 0.977 - 1.027 | 0.9029 |
| **KPS ≥ 80** | 0.979 | 0.963 - 0.994 | 0.0069 | 0.952 | 0.922 - 0.983 | 0.0024 |
| **Dose ≥ 45 Gy** | 2.405 | 1.503 - 3.851 | 0.0003 | 1.658 | 0.638 - 4.312 | 0.2994 |
| **Total Resection** | 0.665 | 0.420 - 1.052 | 0.0811 | 0.765 | 0.304 - 1.924 | 0.5692 |
| **Tumor Size** | 1.006 | 0.992 - 1.019 | 0.4214 | 0.996 | 0.969 - 1.023 | 0.7719 |
| **Multifocal Disease** | 1.435 | 0.940 - 2.191 | 0.0945 | 1.164 | 0.466 - 2.905 | 0.7451 |
| **Tumor Mutational Burden** | 1.047 | 0.936 - 1.171 | 0.4183 | 1.117 | 0.991 - 1.258 | 0.0693 |
| **MGMT Methylation** | 0.399 | 0.211 - 0.756 | 0.0048 | 1.005 | 0.404 - 2.496 | 0.9920 |
| **CDKN2A Deletion** | 1.479 | 0.953 - 2.297 | 0.0809 | 1.554 | 0.666 - 3.626 | 0.3079 |
| **CDKN2B Deletion** | 1.415 | 0.926 - 2.162 | 0.1082 | 3.373 | 1.346 - 8.452 | 0.0095 |
| **MTAP Deletion** | 1.423 | 0.930 - 2.177 | 0.1040 | 1.165 | 0.419 - 3.245 | 0.7694 |
| **PTEN Alteration** | 1.226 | 0.802 - 1.875 | 0.3461 | 0.962 | 0.413 - 2.240 | 0.9277 |
| **EGFR Gain** | 0.852 | 0.556 - 1.308 | 0.4646 | 1.086 | 0.458 - 2.574 | 0.8510 |
| **TP53 Alteration** | 1.033 | 0.657 - 1.624 | 0.8882 | 1.012 | 0.358 - 2.864 | 0.9819 |
| **NF1 Alteration** | 0.969 | 0.554 - 1.696 | 0.9135 | 2.804 | 0.888 - 8.856 | 0.0788 |
| **CDK4 Deletion** | 1.090 | 0.560 - 2.121 | 0.7995 | 0.743 | 0.217 - 2.549 | 0.6370 |
| **EGFR vIII Exon 2-7 Deletion** | 0.822 | 0.454 - 1.488 | 0.5176 | 1.016 | 0.231 - 4.476 | 0.9833 |
| **PIK3CA Alteration** | 1.322 | 0.716 - 2.439 | 0.3719 | 2.011 | 0.580 - 6.969 | 0.2704 |
|  |  |  |  |  |  |  |
